# 1-year postoperative follow-up is adequate for arthroscopic Bankart repair: a comparison between 1-year and 2-year postoperative patient-reported outcome scores

**DOI:** 10.1016/j.jseint.2025.01.011

**Published:** 2025-02-07

**Authors:** Jun-Hao Tan, Evan Teo, V Prem Kumar, Keng Soon Poh, Joel Louis Lim

**Affiliations:** a Department of Orthopaedic Surgery, National University Hospital, National University Health System, Singapore, Singapore; b Department of Orthopaedic Surgery, Yong Loo Lin School of Medicine, National University of Singapore, Singapore, Singapore

**Keywords:** Arthroscopic, Bankart, Outcome scores, Follow-up, MCID, PROMs

## Abstract

**Background:**

The optimal follow-up duration for arthroscopic Bankart repair remains unclear. This study hypothesized that a 1-year postoperative follow-up is adequate for patients undergoing this procedure.

**Methods:**

A retrospective cohort study was conducted from 2009 to 2020, including consecutive patients who underwent arthroscopic Bankart surgery at a university hospital. Seven different patient-reported outcome measures (PROMs) were recorded for all patients at 4 time points: preoperatively, 6 months, 1 year, and 2 years postoperatively. The time to achieve the minimally clinically important difference for the different PROMs was also recorded.

**Results:**

The study included 181 patients. Across all 7 PROMs, improvements exceeded the minimally clinically important difference, with maximal improvement observed by 1 year postoperatively. The complication rate was low (0.55%), and no redislocations occurred. Outcome scores showed no significant difference between 1-year and 2-year follow-ups.

**Conclusion:**

We conclude follow-up up to 1-year postarthroscopic Bankart repair is adequate for recreational athletes. These findings suggest potential optimization of health-care resources by reducing unnecessary clinic visits.

Bankart repair has superior outcomes for traumatic shoulder dislocation as it leads to lower rates of recurrent dislocation and reduces the need for further surgery as compared to conservative treatment.^9^^,^^16^^,^^17^ Over the years, arthroscopic repair of labral tears has gained popularity due to its lower complication rates compared to open surgery.^15^^,^^24^Arthroscopic interventions have also allowed return to preinjury level of sports in 88% of patients in a report by Memon et al.^11^

While it is a successful surgery, adverse outcomes such as nerve injury,^19^ infection,^17^^,^^21^ re dislocation^2^^,^^15^ and degenerative changes in the shoulder^18^ need to be recorded and this mandates a duration of follow-up to detect them. The exact duration of follow-up is, however, unclear. The usual follow-up period required would be 2-3 years,^6^^,^^8^^,^^10^ a duration commonly required by scientific publications for outcome evaluation. The rationale for this duration is also not defined.

A systematic review by Abouali et al^1^ reported series averaging 37.4 months of follow-up following revision arthroscopic Bankart repair for failed surgery. The reason for this duration is unclear. Hayashida et al^14^ followed patients for an average of 40 months. However Sacchetti et al^22^ reported only a 2.5 year follow-up outcomes for all suture anchor repair of labral tears. Shibata et al^23^ reported a 67.5 month follow-up while van Gastel et al^26^ followed patients for 13.1 years and reported a 10% redislocation rate after surgery. Bauer et al^4^ reported an average of 14 years followed-up, and detected glenohumeral osteoarthritic changes in 28.1% of patients.

Clinical follow-up is required to detect complications that develop after surgery. While a minimum of 2 years of follow-up is required for academic publications, there is no clear rational reason for this. Clinical workload and resource utilization are increased with each extra clinical visit without defined patient benefits. Consequently, there exists a critical need to optimize follow-up following any surgical procedure, including arthroscopic Bankart repairs, without compromising patient care and at the same time not overloading clinical service provision.

This study postulates that a 1-year postoperative surveillance period suffices for both clinical evaluation and research endeavors. Our primary objective was to compare outcome scores at the 1-year and 2-year postoperative marks, while our secondary aim sought to delineate the timing of maximal improvement in outcome scores during the postoperative period. Our hypothesis is that there would be no differences in outcome scores between the 2 periods and maximal improvement in outcomes would be reached by 1 year postsurgery.

## Methodology

### Study design

This retrospective cohort study was conducted at a single university hospital, spanning 21 years from 2009 to 2020.

The study included consecutive patients with documented anterior shoulder dislocation and magnetic resonance imaging (MRI)-confirmed labral tear who underwent arthroscopic Bankart repair. Patients undergoing additional procedures such as remplissage for Hill Sachs lesions were excluded, alongside those with prior shoulder surgeries or concurrent biceps, acromioclavicular joint, or rotator cuff surgeries. Individuals with glenoid bone loss exceeding 13% and those without a minimum 2-year follow-up were also excluded to ensure cohort homogeneity.

### Surgical indications

Patients experiencing first-time dislocations were managed conservatively with physiotherapy, focused on rotator cuff, deltoid, and periscapular muscle strengthening exercises. Surgical intervention was considered for those with recurrent instability. Early surgery was advised for individuals in high-risk occupations necessitating overhead use of the arm and for competitive athletes.

### Surgical technique

Surgery was carried out using standard previously described surgical techniques (4, 18). Postoperatively, the shoulder was splinted in a sling for 3 weeks, followed by physiotherapy focused on gaining range of motion followed by strength training. Return to sports was permitted when full motion and strength were achieved.

### Data collection

Demographic details, injury mechanisms, preoperative MRI findings and surgical details were noted. All postoperative complications, including infection, redislocation, subluxation, neurovascular injury, and stiffness, were also recorded. At each time points in the follow-up patients had the anterior apprehension maneuver performed and any apprehension was recorded. The return to daily activity and especially sports participation was also noted

Patient-reported outcome measures (PROMs) were recorded at 4 distinct time points: preoperatively, 6-months, 1-year, and 2 -year postoperatively. The choice of PROMs evolved over time in response to the COVID-19 pandemic's impact on health-care delivery methods. Before 2018, the Constant score, American Shoulder and Elbow Surgeons Shoulder score (ASES), and 36-Item Short Form Survey (SF-36) were employed. Post-2019, to minimize in-person consultations, the Oxford Shoulder Score (OSS), the Oxford Shoulder Instability Score (OSIS), EQ-5D, and QuickDASH score were favored due to their suitability for remote administration.

The assessment of improvement in PROMs was benchmarked against established minimally clinically important difference (MCID) values derived from existing literature. Noteworthy MCID values included 15.5 for ASES,^4^ 6.0 for OSS, 8.6 for OSIS,^26^ 0.21 for EQ-5D,^14^ and 10.0 for QuickDASH. In the absence of a defined MCID for SF-36 specific to arthroscopic Bankart repair or similar shoulder surgeries, a value of 4.0 was adopted, extrapolated from studies on surgery for degenerative cervical myelopathy.^3^The Constant score's threshold score is 68.0 at 6 months and 84.0 at 12 months postoperatively.^25^

### Statistical analysis

Statistical analysis was performed using Statistical Package for Social Science version 20 (version 20.0; IBM Corp, Armonk, NY, USA). Categorical variables are represented as frequencies with percentages and continuous variables as means with standard deviations. Univariate analysis was performed using chi-squared test and paired T-test. Statistical significance was taken as *P* < .05.

### Ethical consideration

Institutional review board approval was obtained prior to the commencement of the study.

## Results

A total of 181 patients, comprising 166 males (91.7%) and 15 females (8.3%) were studied. The mean age at presentation was 25.8 ± 9.9 years, with the mean age of initial shoulder dislocation noted at 22.1 ± 9.3 years. A traumatic injury resulted in all first-time dislocations, predominantly affecting casual sportspersons engaged in weekend activities, with basketball being the most common sport involved followed by soccer. A few patients sustained work-related injuries. The majority of patients were of Chinese ethnicity (75.1%), followed by Malays (12.7%) and Indians (8.3%).). On average, patients experienced 3.3 ± 4.6 shoulder dislocations before presentation, with a mean interval of 53.6 months (range: 1-204 months) from the first dislocation to presentation. Joint laxity was present in 29.8% of cases. All patients displayed anterior inferior labral tears and Hill Sachs lesions on MRI. The average time from presentation to surgery was 6.4 months (range 1-26 months), with an average of 3 anchors used during surgical intervention.

## Patient-reported outcome scores

The study recorded the Constant score, ASES, and SF-36 for 98 patients, while the OSS, OSIS, QuickDASH score, and EQ-5D were recorded for 83 patients. Statistical analysis indicated no significant difference between these two patient groups regarding age (*P* = .889), gender (*P* = .423), or hand dominance (*P* = 1.00).

### Constant score

**Figure 1:**
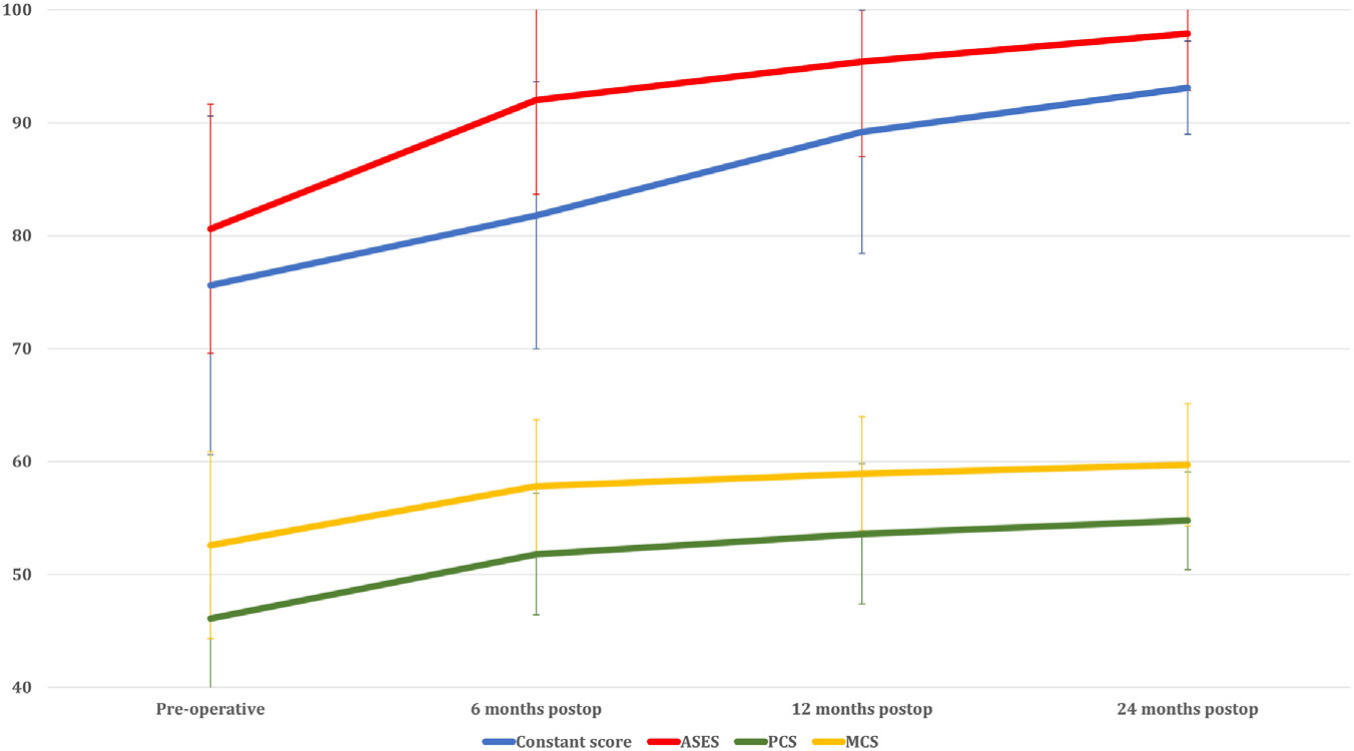
Postoperative improvement of Constant score, ASES, and SF-36. *ASES*, American Shoulder and Elbow Surgeons Shoulder score; *SF-36*, 36-Item Short Form Survey.

The Constant score had a statistically significant improvement from 75.6 ± 13.9 preoperatively to 81.8 ± 12.1 at 6 months postoperatively (*P* = .031) and further improved to 89.2 ± 11.3 at 12 months postoperatively (*P* = .044). However, there was no statistically significant difference observed between 12 months and 24 months postoperatively, with the score plateauing at 93.1 ± 4.1 (*P* = .551) (Table I) (Fig. 1).

**Table I:** Pre and post-operative Constant score, ASES, PCS, and MCS scores.

	Constant score	ASES	PCS	MCS
Preoperative	75.6 ± 13.9	80.6 ± 10.6	46.1 ± 6.5∗	52.6 ± 8.1
6 months postop	81.8 ± 12.1∗	92.0 ± 8.4∗	51.8 ± 5.0∗	57.8 ± 5.0∗
12 mo postop	89.2 ± 11.3∗	95.4 ± 8.9∗	53.6 ± 6.2	58.9 ± 5.1
24 mo postop	93.1 ± 4.1	97.9 ± 5.0	54.8 ± 4.3	59.7 ± 5.4

*Postop*, postoperative; *MCS*, Mental Component Summary; *PCS*, Physical Component Summary.

∗ Indicates a statistically significant improvement as compared to preoperative outcome score.

### ASES score

The ASES score showed a statistically significant improvement from 80.6 ± 10.6 preoperatively to 92.0 ± 8.4 at 6 months postoperatively (*P* < .001), with a further increase to 95.4 ± 8.9 at 12 months postoperatively (*P* = .028). However, there was no statistically significant difference observed between 12 months and 24 months postoperatively, with the score at 97.9 ± 5.0 (*P* = .865) (Table I) (Fig. 1).

### Physical and mental component score of SF-36

The physical component score of SF-36 exhibited a statistically significant improvement from 46.1 ± 6.5 preoperatively to 51.8 ± 5.0 at 6 months postoperatively (*P* < .001). However, there was no statistically significant difference observed between 6 months to 12 months postoperatively (*P* = .071), and from 12 months (53.6 ± 6.2) to 24 months (54.8 ± 14.3) postoperatively (*P* = .713). The mental component score of SF-36 demonstrated a statistically significant improvement from 52.6 ± 8.1 preoperatively to 57.8 ± 5.0 at 6 months postoperatively (*P* < .001). However, there was no statistically significant difference observed between 6 months to 12 months postoperatively (58.9 ± 5.1, *P* = .105), and from 12 months to 24 months postoperatively (59.7 ± 5.4,) (*P* = .933) (Table I) (Fig. 1).

### Oxford Shoulder Score

**Figure 2:**
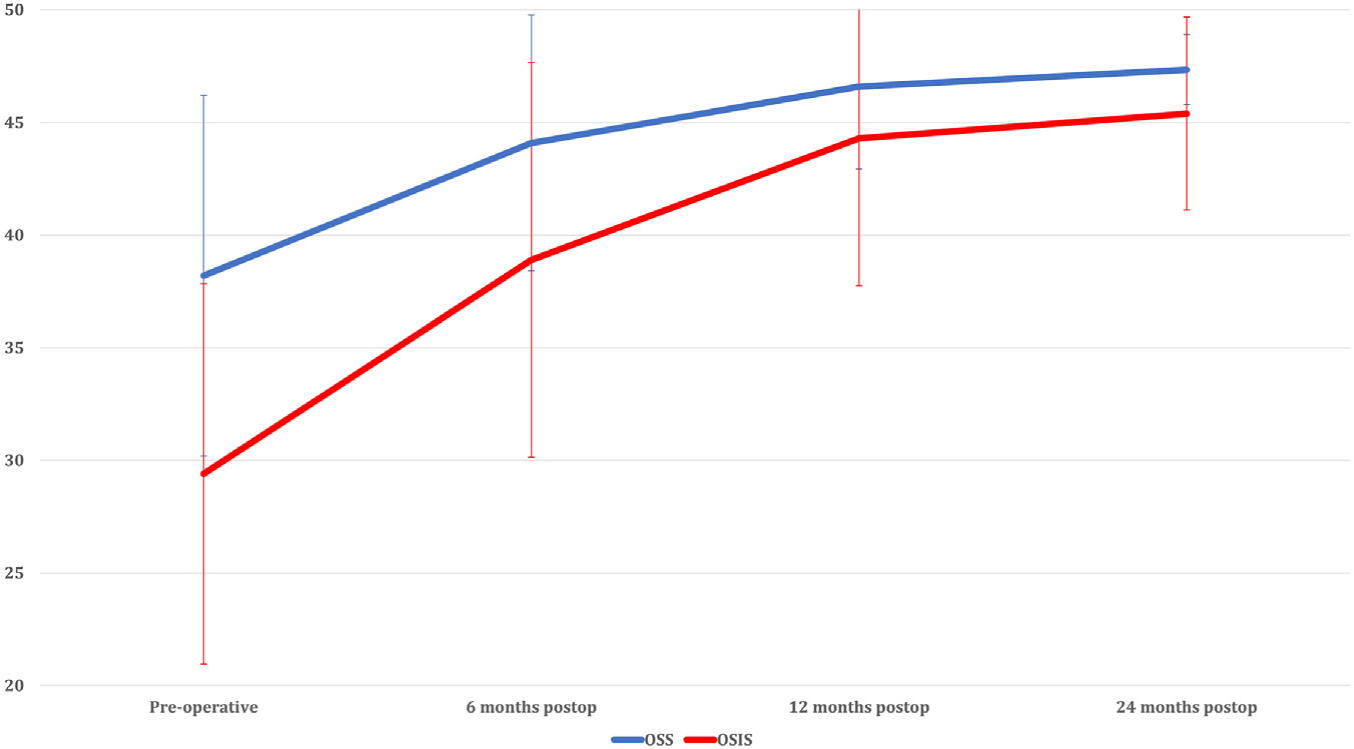
Postoperative improvement of OSS and OSIS. *OSS*, Oxford Shoulder Score; *OSIS*, Oxford Shoulder Instability Score.

The OSS displayed a statistically significant improvement from 38.2 ± 7.9 preoperatively to 44.1 ± 5.1 at 6 months postoperatively (*P* < .001), and further to 46.6 ± 3.0 at 12 months postoperatively (*P* = .031). However, there was no statistically significant difference observed between the scores at 12 months and 24 months (47.3 ± 1.6) postoperatively (*P* = .834) (Table II) (Fig. 2).

**Table II:** Pre and post-operative OSS and OSIS scores.

	OSS	OSIS
Preoperative	38.2 ± 7.9	29.4 ± 8.7
6 mo postop	44.1 ± 5.1∗	38.9 ± 8.0∗
12 mo postop	46.6 ± 3.0∗	44.3 ± 5.0∗
24 mo postop	47.3 ± 1.6	45.4 ± 4.3

*Postop*, postoperative; *OSS*, Oxford Shoulder Score; *OSIS*, Oxford Shoulder Instability Score.

∗ Indicates a statistically significant improvement as compared to preoperative outcome score.

### Oxford Shoulder Instability Score

The OSIS demonstrated a statistically significant improvement from 29.4 ± 8.7 preoperatively to 38.9 ± 8.0 at 6 months postoperatively (*P* < .001), and further to 44.3 ± 5.0 at 12 months postoperatively (*P* = .004). However, no statistically significant difference was observed between the scores at 12 months and 24 months postoperatively (45.4 ± 4.3; *P* = .901) (Table II) (Fig. 2).

### QuickDASH

The disability component of the QuickDASH exhibited a statistically significant improvement from 50.0 ± 17.0 preoperatively to 35.0 ± 11.4 at 6 months postoperatively (*P* < .001), and further to 29.4 ± 8.4 at 12 months postoperatively (*P* < .001). However, no statistically significant difference was observed between the scores at 12 months and 24 months postoperatively (26.4 ± 3.8; *P* = .712).

The optional “work” module of the QuickDASH demonstrated a statistically significant improvement from 71.7 ± 38.0 preoperatively to 43.7 ± 18.5 at 6 months postoperatively (*P* < .001), and further to 39.9 ± 17.2 at 12 months postoperatively (*P* < .001). However, no statistically significant difference was observed between the scores at 12 months and 24 months postoperatively (33.9 ± 4.3; *P* = .117).

**Figure 3:**
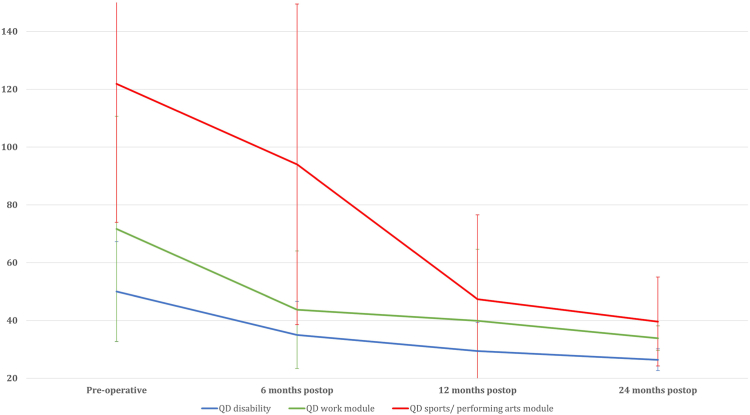
Postoperative improvement of QuickDASH scores.

The optional “sports/performing arts” module of the QuickDASH showed a statistically significant improvement from 121.9 ± 48.5 preoperatively to 94.0 ± 53.6 at 6 months postoperatively (*P* < .001), further improving to 47.4 ± 29.3 at 12 months postoperatively (*P* < .001), and 39.6 ± 15.7 at 24 months postoperatively (*P* = .029) (Table III) (Fig. 3).

**Table III:** Pre and post-operative QD disability, QD work module, QD sports/performing arts module.

	QD disability	QD work module	QD sports/performing arts module
Preoperative	50.0 ± 17.0	71.7 ± 38.0	121.9 ± 48.5
6 mo postop	35.0 ± 11.4∗	43.7 ± 18.5∗	94.0 ± 53.6∗
12 mo postop	29.4 ± 8.4∗	39.9 ± 17.2∗	47.4 ± 29.3∗
24 mo postop	26.4 ± 3.8	33.9 ± 4.3	39.6 ± 15.7∗

*Postop*, postoperative.

∗ Indicates a statistically significant improvement as compared to preoperative outcome score.

### EQ-5D

**Figure 4:**
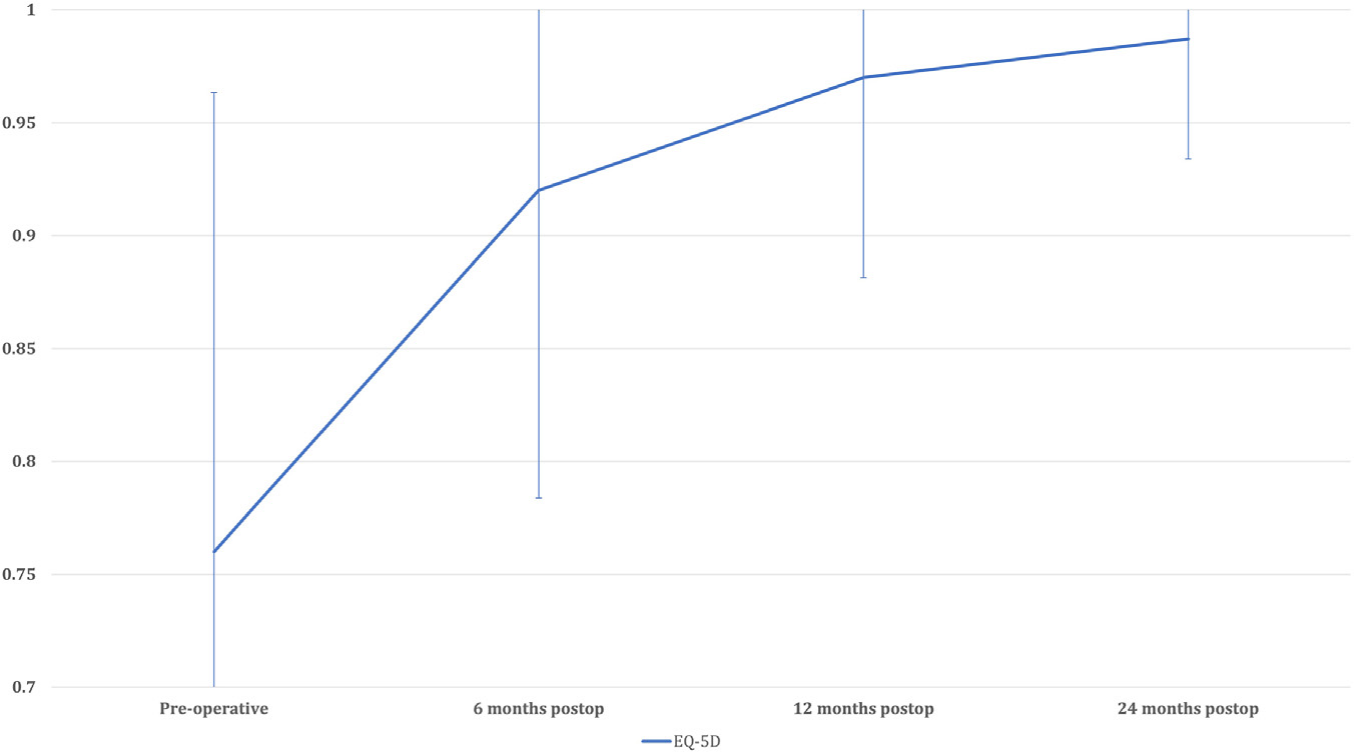
Postoperative improvement of EQ-5D.

The EQ-5D exhibited a statistically significant improvement from 0.76 ± 0.2 preoperatively to 0.92 ± 0.14 at 6 months postoperatively (*P* < .001), and further to 0.97 ± 0.08 at 12 months postoperatively (*P* = .002). However, there was no statistically significant difference observed between the scores at 12 months and 24 months postoperatively (1.0 ± 0.05; *P* = 1.00) (Table IV) (Fig. 4).

**Table IV:** Pre and post-operative EQ-5D.

	EQ-5D
Preoperative	0.76 ± 0.2
6 mo postop	0.92 ± 0.1∗
12 mo postop	0.97 ± 0.08∗
24 mo postop	1.0 ± 0.05

*Postop*, postoperative.

∗ Indicates a statistically significant improvement as compared to preoperative outcome score.

For all 7 PROMs included in this study, the improvement surpassed both the MCID and the threshold scores. For the Physical and Mental component scores of SF-36, the OSIS, and QuickDASH scores including the work and sports/performing arts modules, the average improvement in outcome scores exceeded the MCID at 6 months postoperatively. For the Constant score, the mean score exceeded the threshold score at both 6 months and 12 months postoperatively. For the ASES, OSS, and EQ-5D, the mean improvement in outcome scores reached the MCID at 12 months postoperatively.

### Postoperative complications

One patient developed a superficial wound infection 2 weeks after surgery that resolved uneventfully with oral antibiotics. Twenty patients had a positive anterior apprehension test. In 5 patients, it was noted at the 6 months postoperatively. The remaining 15 demonstrated apprehension at 1-year follow-up. No further investigations were carried out as the patients were not keen on any surgical interventions and were happy to pursue a further course of physiotherapy.

## Discussion

Our study substantiates the hypothesis that there are no significant differences in PROMs between 1 and 2 years postarthroscopic Bankart repairs for recurrent shoulder dislocations, except for the “sports/performing arts” module of the QuickDASH, which showed a modest yet statistically significant improvement. By the 6-month postoperative mark, improvements surpassed the MCID and threshold scores for four PROMs evaluated in this study. By the 12-month mark the remaining three PROMs had achieved the MCID. The authors postulate that for patients with shoulder instability treated surgically regular routine follow-up can stop at 12 months.

The goals of clinical follow-up are to ensure that the patient’s postoperative management was appropriate and to look out for potential complications in the perioperative and early and late postoperative periods. The ability to return to sports of their choice without issues is also a major consideration.

Monitoring immediate postoperative complications focuses primarily on infections and nerve injuries that can impede functional recovery following surgery. Our study reported a solitary case of superficial wound infection among 181 patients, occurring within 2 weeks postsurgery and did not affect patient reported outcomes subsequently. Similarly, low infection rates have also been observed in other studies.^17^^,^^21^ Bitar et al^5^ reported no infections in 19 patients followed up for 84 months. These findings suggest that infections are more likely to manifest early in the postoperative period, obviating the need for long-term follow-ups.

Owens et al reported a nerve injury rate of 0.3% in arthroscopic repairs, notably lower than the 2.2% rate observed in open repairs.^20^ Similarly, Hamada et al noted immediate postoperative axillary nerve palsy in only four shoulders out of 2027 undergoing arthroscopic stabilization.^12^ Others have also reported a low surgery related nerve injury.^17^ We did not encounter any cases of postoperative nerve palsy. This suggests that long-term follow-up solely for the detection of this complication is unnecessary, as nerve injuries are typically identified soon after surgery.

Recurrent dislocation following arthroscopic stabilization has varied from 0.4%^19^ to 10% over a 13-year follow-up period.^2^ We had no cases of frank dislocations in our series although 20 of our patients had a positive anterior apprehension test at various time points during follow-up. None of them required revision surgery.

Most re dislocations occur within the first year following surgery. Virk et al in a study of 58 patients reported 7 clinical failures with a mean time to recurrence observed at an early stage (12.6 months).^27^ Shibata et al followed 100 patients (102 shoulders) postarthroscopic Bankart repair for an average duration of 67.5 months, noting redislocation in 9 shoulders, of which 7 experienced this complication within the first year.^23^ An unaddressed large Hill Sachs lesion and insufficient anchors were identified as contributory to this complication. The latter was also identified as a risk factor for recurrence by Hayashida et al.^14^ MacDonald et al also reported an 18% redislocation rate in 54 patients followed for 24.3 months and attributed the failure to insufficient attention to a significant Hill Sachs lesion.^19^ Most re dislocations will occur early, with, of even without a traumatic event, from failure to address bony and soft tissue defects in the shoulder at the first surgery.

We like to emphasize that a traumatic event would be required for the shoulder to dislocate if all pathologies were addressed at the first surgery. Shibata et al reported 9 redislocations occuring after a traumatic event in a report of 100 patients.^23^ In addition, Cho et al demonstrated a higher failure rate in collision sports compared to noncollision sports following arthroscopic surgery.^7^ Pearce et al reported a 12.9% rate of recurrent dislocation after arthroscopic shoulder stabilization, all linked to major traumatic events.^21^ Abouali et al also concluded that the most common cause for the failure of the primary surgery was a new traumatic event.^1^ These studies underscore the importance of accurately defining surgical failure and the role of traumatic events in recurrent dislocations postarthroscopic stabilization. The latter is vital to define failure and this was lacking in the report by Zimmerman et al who reported instability in 41.7% of patients followed for 16 years after arthroscopic Bankart repair.^29^ The cause of the failures was not clear in that report.

We conclude that the most common cause for failure of the primary surgery is unaddressed bony and soft tissue defects at the time of the first surgery and this is likely to occur early.^5^^,^^13^ No long term follow-up is required to identify this group of patients. And secondly a traumatic event is the likely cause of failure if a proper repair was carried out at the first surgical exercise.^3^^,^^5^^,^^6^^,^^28^This unfortunately can occur at any time point after repair, even years postsurgery and hence a follow-up time cannot be defined.

To maintain homogeneity within our patient cohort, we excluded cases with significant glenoid bone loss and engaging Hill-Sachs lesions. We addressed joint laxity with appropriate capsular plication where needed. We had no dislocations or subluxations during follow-up in our series.

Our study population primarily consisted of casual sports enthusiasts who were receptive to counselling regarding the risks associated with returning to high-contact sports following shoulder surgery. This emphasis on patient education and cautious return to activities contributed to favorable outcomes, in our cohort.

Harris et al's 11-year follow-up revealed that 26% of patients who underwent arthroscopic Bankart repair developed postoperative osteoarthritis.^13^ Similarly, Bauer et al's 14-year follow-up reported a 28.1% incidence of glenohumeral osteoarthritis.^4^ These studies analyzed surgeries conducted over a decade ago, characterized by a less aggressive approach to stabilizing dislocating shoulders. Patients experienced multiple dislocations before surgical intervention, leading to significant chondral damage presurgery and subsequent osteoarthritis. In contrast, Carli et al's study demonstrated that early surgical stabilization significantly reduced osteoarthritis development compared to nonoperative management.^9^ Lee et al found a 15% incidence of osteoarthritis among patients with untreated recurrent posterior shoulder instability during a minimum 5-year follow-up.^18^ In contrast, Chapus et al's report indicated only mild osteoarthritis (Samilson Grade 1) in 3 out of 21 shoulders that underwent early surgical intervention for first-time dislocation at the 10-year follow-up.^6^ With a proactive approach involving early surgical intervention, particularly in high-risk patients, we can potentially minimize the occurrence of osteoarthritis. A long-term follow-up to detect this rare and likely minimally symptomatic complication may not be warranted.

Many studies reporting the cost of shoulder surgery primarily focus on expenses incurred during the initial surgical procedure and immediate postoperative period, neglecting to delve into the costs associated with follow-up visits.^7^^,^^23^ By potentially reducing subsequent outpatient clinic visits, we could likely reduce costs, alleviate clinical burdens, and mitigate logistical challenges linked with extended follow-ups.

In our setting, we achieved successful 1- and 2-year follow-ups for all patients by emphasizing the importance of regular postsurgical visits to monitor for early complications. The access to hospital visits was convenient in our small city-state. We are aware that young patients embarking on new careers might be reluctant to make hospital visits especially if they are asymptomatic. Our study indicates that clinic visits can potentially be reduced further from the usual 2-year period. Our findings also suggest that a minimum 2-year follow-up, even for research purposes, may also not be essential as optimal outcomes postsurgery were achieved within 1 year. Longer follow-up even beyond 2 years may, however, be necessary for professional athletes managed by team physicians to detect reinjuries and to institute treatment.

## Strengths and limitations

A complete follow-up of all the patients over the 2-year period is a strength of this study. However we analyzed two cohorts of patients at different time points using two sets of PROMs. We did not delve into intraoperative factors such as the extent of labral tears or specifics of repair types. In addition, no follow-up MRI assessments were conducted. The retrospective nature of our study also posed inherent limitations, and the series was limited to patients undergoing only a Bankart repair and who were predominantly casual sportsmen.

## Conclusion

Our study supports the idea that a 1-year follow-up period following arthroscopic Bankart repair is adequate for both clinical assessment and research purposes for the casual sportsmen. No significant differences in almost all PROMs were noted between the 1-year and 2-year assessment points. MCIDs were reached in all PROMs by 1 year. Complications, if present, are typically identified early in the postoperative phase, rendering long-term follow-ups primarily for complication detection unnecessary. We also emphasize that the primary cause of surgical failure post-Bankart repair is a new traumatic incident or unaddressed glenohumeral deficiencies. However, it's important to note that our study is retrospective, and future large prospective studies are necessary to validate and expand upon our findings.

## Disclaimers:

Funding: No funding was disclosed by the authors.

Conflict of interest: The authors, their immediate families, and any research foundation with which they are affiliated have not received any financial payments or other benefits from any commercial entity related to the subject of this article.

## References

[1] Abouali J.A., Hatzantoni K., Holtby R., Veillette C., Theodoropoulos J. (2013). Revision arthroscopic Bankart repair. Arthroscopy.

[2] Alkaduhimi H., Connelly J.W., van Deurzen D.F.P., Eygendaal D., van den Bekerom M.P.J. (2021). High variability of the definition of recurrent glenohumeral instability: an analysis of the current literature by a systematic review. Arthrosc Sports Med Rehabil.

[3] Badhiwala J.H., Witiw C.D., Nassiri F., Akbar M.A., Jaja B., Wilson J.R. (2018). Minimum clinically important difference in SF-36 scores for use in degenerative cervical myelopathy. Spine.

[4] Bauer A., Engel G., Huth J., Mauch F. (2023). Fourteen years of follow-up after first arthroscopic Bankart repair in athletes: functional outcomes and magnetic resonance imaging findings. J Shoulder Elbow Surg.

[5] Bitar A.C., Fabiani M.C., Ferrari D.G., Garofo A.G.P., Schor B., Zorzenoni F.O. (2021). Clinical and functional outcomes of the remplissage technique to repair anterior shoulder dislocation: average 7 years of follow-up. Musculoskelet Surg.

[6] Chapus V., Rochcongar G., Pineau V., Salle de Chou É., Hulet C. (2015). Ten-year follow-up of acute arthroscopic Bankart repair for initial anterior shoulder dislocation in young patients. Orthop Traumatol Surg Res.

[7] Cho N.S., Hwang J.C., Rhee Y.G. (2006). Arthroscopic stabilization in anterior shoulder instability: collision athletes versus noncollision athletes. Arthroscopy.

[8] Crall T.S., Bishop J.A., Guttman D., Kocher M., Bozic K., Lubowitz J.H. (2012). Cost-effectiveness analysis of primary arthroscopic stabilization versus nonoperative treatment for first-time anterior glenohumeral dislocations. Arthroscopy.

[9] De Carli A., Vadalà A.P., Lanzetti R. (2019). Early surgical treatment of first-time anterior glenohumeral dislocation in a young, active population is superior to conservative management at long-term follow-up. Int Orthop.

[10] Evans J., Dattani R., Ramasamy V., Patel V. (2018). Responsiveness of the EQ-5D-3L in elective shoulder surgery: does it adequately represent patient experience?. J Orthop Surg.

[11] Foong W.S., Zeng G.J., Goh G., Hao Y., Lie D.T.T., Chang P.C.C. (2022). Determining the minimal clinically important difference on the Oxford shoulder instability score in patients undergoing arthroscopic bankart repair for shoulder instability. Orthop J Sports Med.

[12] Hamada H., Sugaya H., Takahashi N., Matsuki K., Tokai M., Ueda Y. (2020). Incidence of axillary nerve injury after arthroscopic shoulder stabilization. Arthroscopy.

[13] Harris J.D., Gupta A.K., Mall N.A. (2013). Long-term outcomes after Bankart shoulder stabilization. Arthroscopy.

[14] Hayashida K., Yoneda M., Nakagawa S., Okamura K., Fukushima S. (1998). Arthroscopic Bankart suture repair for traumatic anterior shoulder instability: analysis of the causes of a recurrence. Arthroscopy.

[15] Hendawi T., Milchteim C., Ostrander R. (2017). Bankart repair using modern arthroscopic technique. Arthrosc Tech.

[16] Hurley E.T., Manjunath A.K., Bloom D.A. (2020). Arthroscopic bankart repair versus conservative management for first-time traumatic anterior shoulder instability: a systematic review and meta-analysis. Arthroscopy.

[17] Law B.K., Yung P.S., Ho E.P., Chang J.J., Chan K.M. (2008). The surgical outcome of immediate arthroscopic Bankart repair for first time anterior shoulder dislocation in young active patients. Knee Surg Sports Traumatol Arthrosc.

[18] Lee J., Woodmass J.M., Bernard C.D. (2022). Nonoperative management of posterior shoulder instability: what are the long-term clinical outcomes?. Clin J Sport Med.

[19] MacDonald P., McRae S., Old J., Marsh J., Dubberley J., Stranges G. (2021). Arthroscopic Bankart repair with and without arthroscopic infraspinatus remplissage in anterior shoulder instability with a Hill-Sachs defect: a randomized controlled trial. J Shoulder Elbow Surg.

[20] Owens B.D., Harrast J.J., Hurwitz S.R., Thompson T.L., Wolf J.M. (2011). Surgical trends in Bankart repair: an analysis of data from the American board of orthopaedic surgery certification examination. Am J Sports Med.

[21] Pearce S.S., Horan M.P., Rakowski D.R., Hanson J.A., Woolson T.E., Millett P.J. (2023). Knotless all-suture, soft anchor bankart repair results in excellent patient-reported outcomes, high patient satisfaction, and acceptable recurrent instability rates at minimum 2-year follow-up. Arthroscopy.

[22] Sacchetti F., Di Meglio M., Mondanelli N., Bianchi N., Bottai V., Cartei F. (2021). Arthroscopic labral repair with all-suture anchors: a magnetic resonance imaging retrospective study with a 2.5-year follow-up. Med Glas.

[23] Shibata H., Gotoh M., Mitsui Y., Kai Y., Nakamura H., Kanazawa T. (2014). Risk factors for shoulder re-dislocation after arthroscopic Bankart repair. J Orthop Surg Res.

[24] Tahta M., Akmeşe R., Özberk Z., Coşkun O.O., Işik C., Korkusuz F. (2013). Muscle strength and function of shoulders with Bankart lesion after successful arthroscopic treatment: interlimb comparison 24 months after surgery. Arch Orthop Trauma Surg.

[25] Uzun E., Doğar F., Topak D., Güney A. (2021). Comparison of anterior single- and standard two-portal techniques in arthroscopic Bankart repair. Jt Dis Relat Surg.

[26] Van Gastel M.L., Willigenburg N.W., Dijksman L., Lindeboom R., van den Bekerom M.P.J., van der Hulst V.P.M. (2019). Ten percent re-dislocation rate 13 years after the arthroscopic Bankart procedure. Knee Surg Sports Traumatol Arthrosc.

[27] Virk M.S., Manzo R.L., Cote M., Ware J.K., Mazzocca A.D., Nissen C.W. (2016). Comparison of time to recurrence of instability after open and arthroscopic bankart repair techniques. Orthop J Sports Med.

[28] Xu S., Chen J.Y., Hao Y., Chang P.C.C., Lie D.T.T. (2020). Threshold scores for treatment success after arthroscopic bankart repair using Oxford shoulder instability score, constant-murley score, and UCLA shoulder score. J Orthop.

[29] Zimmermann S.M., Scheyerer M.J., Farshad M., Catanzaro S., Rahm S., Gerber C. (2016). Long term restoration of anterior shoulder stability: a retrospective analysis of arthroscopic Bankart repair versus open Laterjet procedure. J Bone Joint Surg Am.

